# Early Detection of Airborne Inoculum of *Nothopassalora personata* in Spore Trap Samples from Peanut Fields Using Quantitative PCR

**DOI:** 10.3390/plants9101327

**Published:** 2020-10-09

**Authors:** Misbakhul Munir, Hehe Wang, Nicholas S. Dufault, Daniel J. Anco

**Affiliations:** 1 Department of Plant and Environment Science, Clemson University, Edisto Research and Education Center, Blackville, SC 29817, USA; mmunir@clemson.edu (M.M.); hehew@clemson.edu (H.W.); 2 Department of Plant Pathology, University of Florida, Gainesville, FL 32611, USA; nsdufault@ufl.edu

**Keywords:** specific primers, *Cercosporidium personatum*, spore collection, *Arachis hypogaea*

## Abstract

A quantitative PCR (qPCR)-assay was developed to detect airborne inoculum of *Nothopassalora personata*, causal agent of late leaf spot (LLS) on peanut, collected with a modified impaction spore trap. The qPCR assay was able to consistently detect as few as 10 spores with purified DNA and 25 spores based on crude DNA extraction from rods. In 2019, two spore traps were placed in two peanut fields with a history of LLS. Sampling units were replaced every 2 to 4 days and tested with the developed qPCR assay, while plots were monitored for symptom development. The system detected inoculum 35 to 56 days before visual symptoms developed in the field, with detection related to environmental parameters affecting pathogen life-cycle and disease development. This study develops the framework of the qPCR spore trap system and represents the initial steps towards validation of the performance of the system for use as a decision support tool to complement integrated management of LLS.

## 1. Introduction

Late leaf spot (LLS), caused by *Nothopassalora personata* (Berk. and M. A. Curtis) U. Braun, C. Nakash., Videira and Crous (teleomorph: *Mycosphaerella berkeleyi* W. A. Jenkins), is one of the most widely distributed and destructive diseases of peanut (*Arachis hypogaea* L.) worldwide and, since the late 1970s, has become the most damaging fungal foliar disease of peanut in the southeastern and Virginia–Carolina regions of the U.S. [1]. Favorable environmental conditions [2,3], susceptible planted cultivars [4], and increased peanut hectarage [5] have contributed to high levels of LLS severity in these region.

Environmental conditions favorable for LLS epidemics, e.g., temperature between 15 and 20 °C and extended periods of high relative humidity (RH ≥ 93% for ≥ 12 h), commonly occur during the growing season in the southeastern and Virginia–Carolina regions of the U.S. [2,6,7,8]. Epidemics of LLS are initiated with production of conidia on peanut residue in the soil following early rains [9]. LLS is primarily spread by airborne conidia, where wind and rain have been demonstrated to play a significant role in their dispersal [10]. Once disseminated to peanut leaves, conidia may germinate, and infection may subsequently occur. Lesions of LLS are dark brown to black and covered with tufts of conidiophores on abaxial sides of leaflets and normally first develop on the oldest leaves nearest the soil surface [1,7,9]. Conidiophores on lesions provide secondary inoculum during the growing season [7]. Under favorable conditions and the absence of control, LLS can result in total defoliation.

Commercial LLS control typically utilizes repeated applications of fungicide. Yield losses due to leaf spot have been reported to approach from 50 to 70% in the absence of fungicide application, while this has been reported to be reduced to 5% with fungicides [7,11,12,13]. Fungicide application for leaf spot control in the southeastern and Virginia–Carolina regions of the U.S. is primarily based on a calendar schedule, starting as early as 30 to 40 days after planting (DAP) and continuing at 10- to 14-day intervals until 14 to 21 days before inversion, making approximately 5–7 fungicide applications within a growing season [7,14,15]. While predictable, the initiation of calendar-based fungicide applications may not, under all field and environmental conditions, always correspond to the actual appearance of inoculum in the field. 

The implementation of inoculum-detection-based fungicide applications has been demonstrated in several pathosystems [16,17,18]. Enhanced control of downy mildew on hop yards was achieved by detection-based applications made 34 days earlier than those prescribed by a calendar schedule [17]. In other instances, applications based on detection reduced unnecessary early season applications as well as the total number of applications for the effective management of powdery mildew on grape [18] and downy mildew on lettuce [16] and hop [17]. The development and use of such systems for peanut LLS management may likewise improve the timing of fungicide application and reduce the potential exposure of *N. personata* populations to active ingredients in the field to assist with resistance management [19].

Fundamental components for detection-based warning systems include an efficient method for sampling (for LLS, the air) and a rapid, specific, and sensitive inoculum detection assay for the pathogen. Spore traps have been used to sample the airborne inoculum of several pathogenic fungi, including *N. personata* [20]. While the detection and quantification of inoculum previously [20,21] examined fungal morphology, this method is often time-consuming, laborious, and requires extensive knowledge of classical taxonomy [22]. Quantitative PCR (qPCR) and its derivations have since become the standard approach to detecting and quantifying pathogen inoculum based on nucleic acids [18,23,24,25,26,27]. Nucleic-acid-based approaches have, furthermore, been paired with air samplers to detect inoculum in several pathosystems [16,23,24,26,28,29,30,31,32]. 

For LLS on peanut, an effective combination of nucleic-acid-based detection specific to *N. personata* and an air sampler has not been developed. Therefore, the objectives of this study were to (i) develop a qPCR with crude DNA extraction procedure suitable for the rapid, specific, and sensitive detection of *N. personata* and (ii) test its capacity for implementation in peanut fields when coupled with a modified impaction spore trap. This study represents the first step toward the ultimate goal of utilizing in-field inoculum detection as a fungicide application decision support tool for the management of LLS on peanut.

## 2. Results

### 2.1. qPCR Optimization

**Figure 1:**
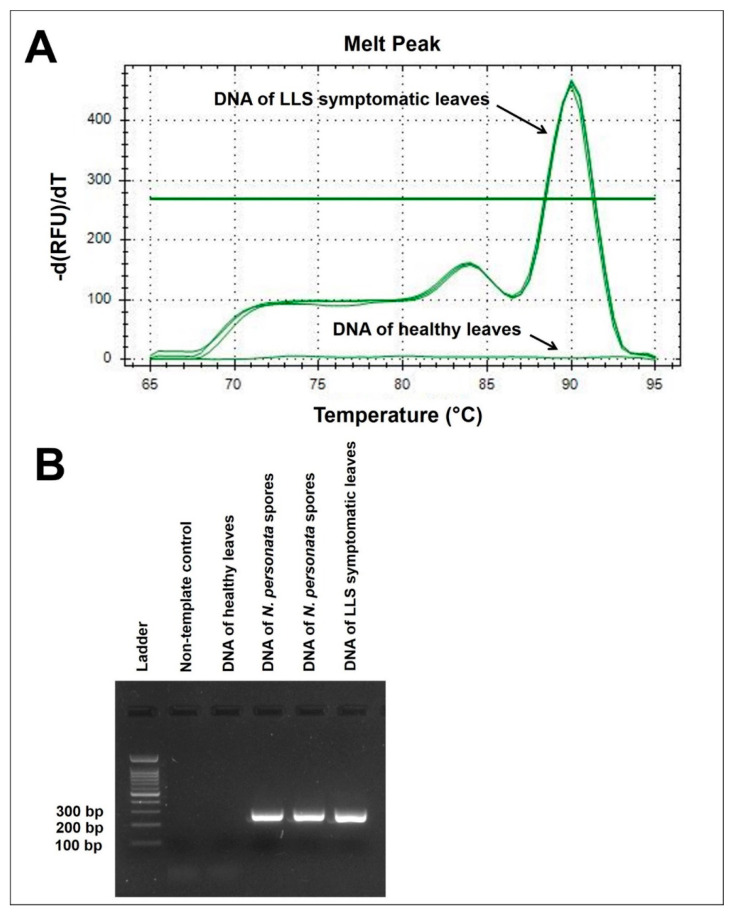
Quantitative Polymerase Chain Reaction (qPCR) assay using primers CP5F-CP5R. (**A**) Melt peak resulting from the qPCR assay. (**B**) Agarose gel electrophoresis of PCR products from the qPCR assay with DNA from healthy peanut leaves, *Nothopassalora personata* spores, and late leaf spot (LLS) symptomatic leaves.

The melt curve of the amplicon showed two peaks (Figure 1A); however, gel electrophoresis revealed a single band of approximately 270 bp (Figure 1B). The shoulder in the melt curve between 83 and 88 °C (Figure S1) suggested the presence of an intermediate state where the DNA is in both double- and single-stranded configurations [33]. 

### 2.2. Amplification Specificity of the qPCR Primers

Amplifications using CP5F-CP5R primers occurred in qPCR with all DNA samples extracted from *N. personata* isolates (collected from nine counties in SC and three counties in FL) and peanut leaves with LLS lesions (Table S1). No amplification occurred in association with DNA extracted from other fungi isolated from peanut leaves, healthy peanut leaves, or leaves with non-LLS symptoms (Figure 1B, Table S1, Figure S2). Fungal DNA samples that yielded a negative qPCR result using CP5F-CP5R primers produced positive amplification when paired with ITS1-ITS4 primers (Table S1). 

No qPCR amplification was observed with air biota samples collected from the fields from prior to planting until 5 weeks after planting (Table S1). Sequence analysis using BLASTn toward the amplicons from the two air samples (field samples) with positive detection showed 100% sequence identity with the ITS sequence of *N. personata* (NR 156379.1 and MF951373.1) and *M. berkeleyi* (AB435066.1 and AY266147.1). 

### 2.3. Detection Limit of the qPCR Assay

**Figure 2:**
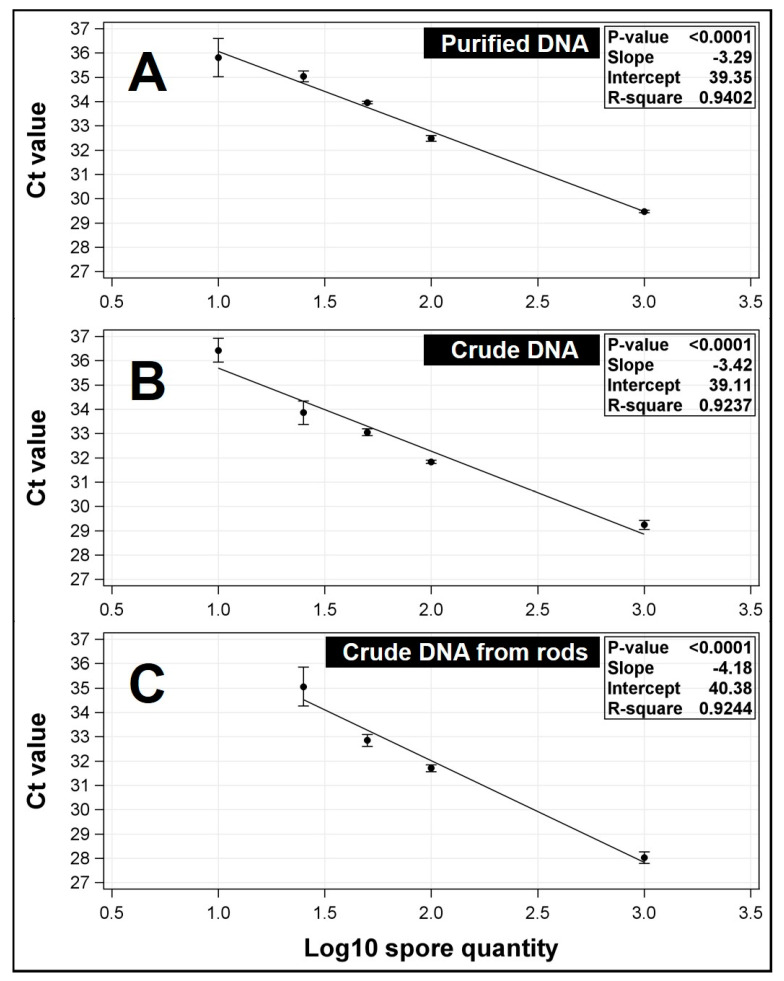
Standard curves of qPCR assay with purified and crude *Nothopassalora personata* DNA. (**A**) Standard curve with purified DNA extracted from 10 to 1000 spores using the commercial DNA extraction kit. Each point corresponds to the average of three independent extractions (± standard error) with three technical replicates per extraction. (**B**) Standard curve with crude DNA extracted from 10 to 1000 spores using crude DNA extraction. (**C**) Standard curve with crude DNA extracted from silicon grease-coated rods artificially inoculated with 25 to 1000 spores using crude DNA extraction. This standard curve was used to estimate number of spores/rods collected in the field.

The optimized qPCR assay consistently amplified DNA from pure spore suspensions at the minimum tested level of 10 spores per sample (Figure 2A,B). Crude and purified DNA resulted in comparable qPCR results (C_t_ values ± SE = 36.43 ± 0.49 and 35.81 ± 0.78, respectively, for 10 spores/sample), particularly when the spores/sample was ≥ 25 (Figure 2A,B). On rods, a 100% detection rate was consistently observed in all crude DNA samples extracted with ≥25 spores/ rod (Figure 2C). Based on these results, a minimum threshold equivalent of 25 spores (C_t_ value = 35.06 ± 0.79) was selected for detection of field samples (Figure 2C). All positive results below this threshold were considered to qualitatively confirm the presence of spores. Linear regression between C_t_ values and Log_10_ spores was significant (*P* < 0.0001) with *R*^2^ = 0.9244 (Figure 2C).

### 2.4. Detection of Airborne Inoculum in Peanut Fields

**Figure 3:**
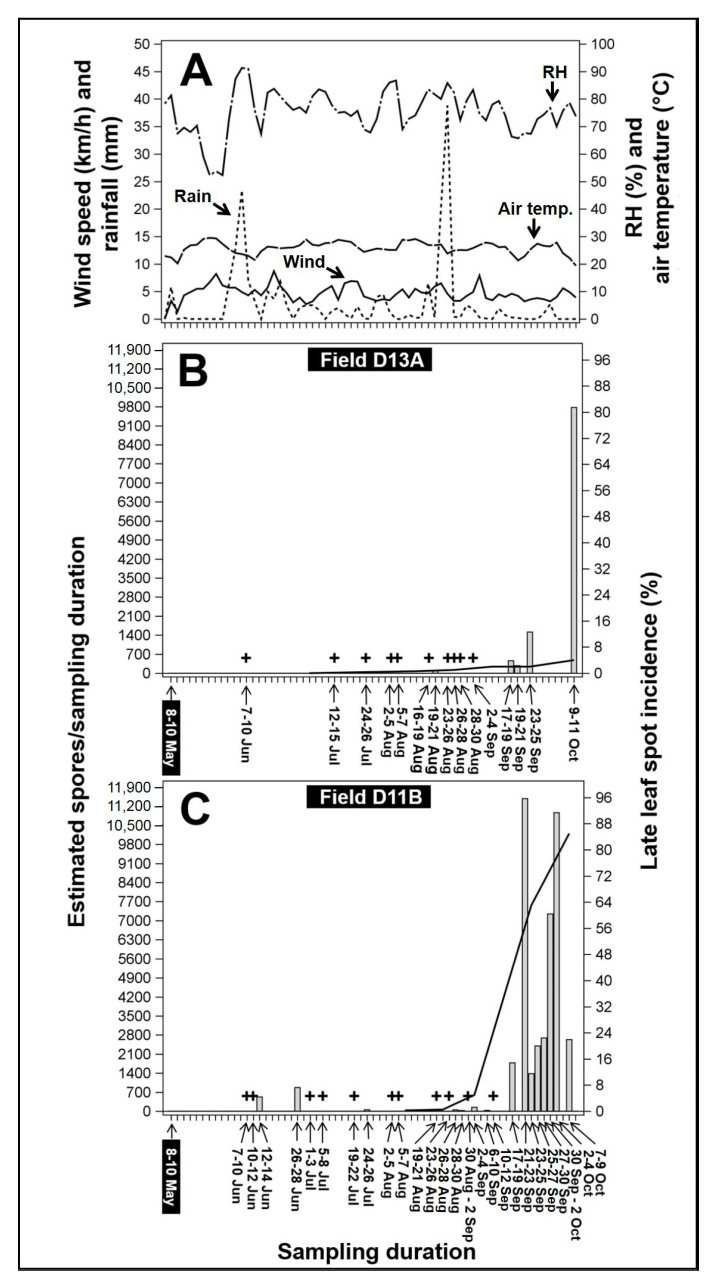
Weather data, inoculum detection, and late leaf spot (LLS) incidence during the 2019 growing season in Blackville, SC. (**A**) Weather data: dashed short line = average accumulated rainfall, solid line = average wind speed, dashed long line = average temperature, and dashed long and short line = average relative humidity (RH). (**B**) Estimated trapped *Nothopassalora personata* spores and LLS incidence in field D13A and (**C**) field D11B. (+) = positive detection below limit of quantification, bars = estimated number of spores collected on the spores/trap per sampling duration, and solid line = % LLS incidence. Magnified bar graph for estimated number of spores <700, is provided in a supplementary figure (Figure S4).

In D13A (Figure S3), air biota samples collected from 16 April to 31 May 2019 did not produce positive amplification following qPCR (Figure 3B) (data from 16 April to 7 May available in Table S1). Samples collected from D11B (Figure S3) in May 2019 similarly did not produce positive amplification (Figure 3C). In a multi-year field study in three geographically different peanut fields in Georgia, Alderman and Nutter (1994) showed that the number of *N. personata* spores trapped by the spore traps within the period prior to the 200th day of the year (prior to the middle of July) was zero or close to zero in most fields and no LLS symptoms were observed until ≥200 day of year (approximately 18 July or after) [2]. While the previous study demonstrated that *N. personata* spore release is uncommon in April and May, the lack of visible LLS symptoms or positive detection in either field during this time in the current study suggest that LLS infections were not developing near the area early in the growing season, and that the primers were specific even in the presence of large quantities of background DNA. The first samples with positive amplification were collected from both fields between 7 to 10 June (Figure 3B,C). Visual LLS symptoms were not apparent within the week of first positive detection. However, LLS symptoms were observed on volunteer peanut in the cotton plot next to D11B, approximately 9–20 m away from the spore trap in D11B (Figure S3). Visual LLS incidence in the volunteer peanut was very low (<1%), while LLS incidence in both D13A and D11B was 0%.

While RH prior to 7 June was relatively low (52–87%), average RH during the first positive detection (7 to 10 June) was higher (91%) and was the highest over the collective sampling duration (Figure 3A). Average air temperature during this initial detection was 23 °C (Figure 3A), with precipitation accumulation averaging 23 mm. Precipitation accumulation during the previous 30 days (8 May to 6 June) was lower, ranging from 0 to 6 mm (Figure 3A). Average wind speed from 7 to 10 June was 4.9 km/h, which was slightly slower compared to that of 1–6 June (6 km/h) (Figure 3A). The first positive detection with the corresponding environmental conditions suggested that the earliest spore production and release event likely occurred during from 7 to 10 June in these fields. Positive detection was more frequent after 17 September (Figure 3A), when average daily temperature ranged from 20 to 27.8 °C and daily RH ranged from 65 to 78%.

The qPCR–spore trap combination detected inoculum before visual observations of symptoms in each field (16 July and 7 August for D13A and D11B, respectively) (Figure 3B,C). Although LLS infections in D13A appeared earlier than in D11B, incidence in D13A over the course of the growing season was overall lower in magnitude (Figure 3B,C). Incidence in D13A started from ~0.2% on 16 July and increased slightly to 4% by 10 October. In D11B, incidence was ~0.25% on 7 August and increased to 85% by 7 October (Figure 3C). Positive detection in each field became more frequent as corresponding LLS incidence increased, being more frequent overall in D11B (Figure 3C). 

## 3. Discussion

This study describes the development of a qPCR-based inoculum detection procedure for *N. personata* and reports its first successful use with a modified impaction spore trap to detect inoculum in peanut fields. Fungal characterization and disease diagnosis using available and conventional in vitro methods (e.g., culturing on media) are hindered for *N. personata* due to its slow growth [34]. Increasing interest in genetic analysis of *N. personata* [35,36] favors the development of molecular approaches to detect, identify and diagnose LLS [36]. 

The developed qPCR procedure was specific to *N. personata*. It efficiently detected all positive samples of *N. personata* from both SC and FL and did not identify false positives among non-target DNA samples tested. Two melting peaks with a single band indicated that the qPCR products melted in multiphase, which could be due to high GC content [33]. As double-stranded DNA starts to melt, a higher temperature is required to melt the more stable (GC-rich) regions. This scenario results in two melting phases where the DNA is in both double- and single-stranded configurations, yielding two qPCR peaks [33].

In addition to being specific, the qPCR procedure facilitated sensitive detection of *N. personata* inoculum. When spores/rod were ≥25, even when potential inhibitors (e.g., silicon grease and metal rods) were present, qPCR paired with crude DNA extraction resulted in comparable C_t_ values to qPCR performed with DNA purified using a commercial kit or crude DNA directly extracted from pure spore suspensions (Figure 2). While the accuracy and sensitivity of nucleic-acid-based detection is largely affected by the quality of the nucleic acids following individual extraction methods [26], optimal extraction further depends on several factors including type of propagule sampled, collection matrix, and the presence of non-target particles [26,37]. DNA fragment size also impacts the sensitivity of detection and quantification efficiency [26,38]. Collectively, these factors could potentially influence detection limits should the procedures developed in the present study be adapted for different fungal species or applications.

The quantification of trapped spores in this study was estimated based on the qPCR standard curve, which was based on artificially inoculated rods in the lab. While useful in understanding theoretical limits of detection, it may not represent the exact number of propagules sampled. The utilization of lab-generated standard curves may bypass several sources of experimental error that otherwise affect DNA extraction and amplification from field samples [39]. The accumulation of inhibitors on the collection matrix during sampling has been reported to negatively affect DNA extraction or PCR efficiency [17]. While silicone grease and metal rods did not appear to affect the efficiency of DNA extraction, we did not quantify potential extraction inhibitors from the field; nevertheless, polymerase and master mixes robust to inhibitors were utilized to limit adverse inhibitor effects. Furthermore, the estimated number of field-caught *N. personata* spores was comparable to that reported by Mallaiah and Rao (1980) [20]: peak daily mean concentration was approximately 1400 spores/cm^2^ at 95 DAP in October 1975 compared to 1163 and 1366 spores/cm^2^ in D13A and D11B, respectively. 

While related, detection and quantification limits are independent assay traits, since detection can be accurate at low spore concentrations while quantification itself may not [26,31]. Accordingly, the scope of the current study emphasized inoculum detection. In the field, although quantitative information on inoculum density would be informative, knowledge of the presence or absence of inoculum is appropriate for initiating control measures early in the growing season and terminating control actions later in the season [17]. Information on the simple presence of inoculum, when inoculum levels are present yet very low, can overestimate potential disease risk (type I error) and increase recommended use of disease control measures (less type II errors). Considering that growers can be risk-averse [40], this approach decreases the more serious situation of not implementing management tactics when they are warranted.

In addition to detecting inoculum (35 to 56 days) prior to visual onset of primary infections or static calendar timings, trap-based detection coincided with favorable environmental conditions for spore production and dispersal and subsequent epidemic development. The first positive detection followed near-optimal temperature and RH for *N. personata* spore production [2]. Prior to this, when temperature and RH were suboptimal for spore production [1,20], detection specificity was not impeded by non-target fungal spores that circulated around the peanut fields. Greater rainfall analogously was associated with the first positive detection compared to earlier samplings, which greatly facilitates spore dissemination and their consequent capacity to be captured [1,10,20]. While wind and rain have been reported to play a significant role in the dispersal of *N. personata* spores (10, 20), rain was more effective at removing conidia of *N*. *personata* than wind [10]. Wadia et al. (1998) associated this greater effectiveness of rain relative to wind in removing *N. personata* conidia with the tap mechanism, in which the vibration of leaves caused by raindrops allows the removal of *N. personata* spores on the abaxial leaf surface [10].

As anticipated, spores were more frequently detected when disease incidence was significant (≥5%; 17 September to 11 October) (Figure 3). During this period, temperature and RH conditions were similar to those reported by Mallaiah and Rao (1980) [20] during periods of increased captures of *N. personata* conidia: average daily temperature from 29 to 30 °C with 65–75% RH. Peak LLS inoculum capture at Blackville in 2019 was between 17 September to 11 October. It is worth noting that 2019 was a year when environmental conditions were, overall, associated with more tempered LLS development across South Carolina [41]. While the calendar-based application suggests initiating fungicide treatment from 30 to 45 DAP (i.e., 23 May to 7 June relative to the planting date of 23 April for both experimental fields in this study), the inoculum sampling system supported the idea to delay fungicide application for both examined fields until from 45 to 48 DAP (first positive detection, 7 to 10 June). This represents a potential saving of one preventative fungicide application under the environmental and background inoculum conditions of the one location year examined. Further field data is necessary to validate the profitability of detection-based fungicide application initiation, as well as the amount of practical notice such a warning system would provide in order to apply fungicide over affected hectarage, which could take several days in the absence of inclement weather limiting field access [42].

Variability between traps was observed both in instances of positive detection and estimated quantities of spores, as has been previously reported [26,31]. Villari et al. (2017) reported large differences in numbers of *Magnaporthe oryzae* spores collected among traps 23 m apart on turfgrass on a given day: >400 in one and <10 spores in another [31]. In the current study, two spore traps were 360 m apart and exhibited similar instances of variability: >5600 spores/day in D11B versus none in D13A between 21 and 23 September. Detection and greater spore numbers were more frequently observed in D11B, where volunteer peanuts with sporulating LLS lesions were more abundantly nearby (~9 to 20 m from the corresponding spore trap) and LLS infections within the field developed earlier. Aside from distance to an inoculum source, many factors have been reported to influence sampling variability among spore traps, including the stochasticity of air turbulence, velocity, and direction in relation to inoculum sources [26,31]. Monthly average wind direction in the experimental fields on June, July, August, September and October 2019 were southwest, southwest, west–southwest, south, and south–southwest, respectively [43]. A higher frequency of positive detection and greater number of trapped spores at D11B may be associated with these wind directions. When the first positive detection was observed, an abundant source of primary inoculum (volunteer peanuts with sporulating LLS lesions) was identified at the cotton field next to D11B (to the east of D11B) (Figure S3). While D13A was located to the north east of D11B (Figure S3), the common west–southwest or south wind directions during increased disease development (August and September) may have facilitated more spores being captured by the trap at D11B compared to that at D13A. 

In general, most peanut plots in D13A and D11B were managed for LLS by the application of chlorothalonil (a contact fungicide) and several systemic fungicides (e.g., prothioconazole plus tebuconazole, penthiopyrad, and thiophanate-methyl) following the standard fungicide application guideline for South Carolina peanut production [12] on a fifteen day interval. Plots within D13A were used for various fungicide trials, which included a relatively limited number of non-sprayed plots. One 0.20-ha trial in the eastern section of D11B did not receive fungicide applications during the growing season, however, LLS lesions in that particular trial were not evident until 7 August. While this non-sprayed test within D11B may have acted as a contributing source for the greater quantity of *N. personata* spores trapped in September and October compared to the trap near D13A, it is unlikely that the non-sprayed trial affected detection events prior to Aug. In addition, cultivars with greater resistance to LLS were planted in D13A compared to those in D11B. These collective factors, in addition to the previously described role of wind direction, may have contributed to the discrepancy in LLS incidence and frequency of detection among the two fields. 

In summary, we developed a qPCR-based inoculum detection method that is sensitive and specific to *N. personata*. We demonstrated its potential to be used with a spore trap to monitor inoculum in peanut fields and documented detection early in the epidemic before development of visual symptoms. This system has capacity as a tool to guide fungicide initiation and potentially improve the efficiency of LLS disease management, with further research capable of estimating the cost-effectiveness and logistical practicality of detection-based fungicide initiation compared to calendar scheduling under varying environmental conditions.

## 4. Materials and Methods

### 4.1. Preparation of LLS Symptomatic Peanut Leaves, N. personata Spores, and Sampling Rods

#### 4.1.1. Preparation of LLS Symptomatic Peanut Leaves for Use in the Initial Development of qPCR Assay

Air-dried and fresh peanut leaves with visible LLS lesions were collected from a peanut field at the Edisto Research and Education Center in Blackville, SC. Leaflets were put in a 2-mL screw cap-tubes (Neptune Screw Cap MicroTubes, Gentaur USA-Genprice Inc, San Jose, CA, USA) and subjected to DNA extraction (see below Section 4.2.1).

#### 4.1.2. Preparation of *N. personata* Spores for Use in the Initial Development of the qPCR Assay

Approximately 15 LLS lesions with visually abundant conidiophores were cut and placed in a 2-mL screw cap-tube. Sterile distilled deionized water (500 µL) was added to the tube prior to being vortexed at 42 Hz for 1 min to release spores. Approximately 500 µL of the resulting spore suspension was transferred to a new 2-mL screw cap-tube and centrifuged at 20,000× *g* for 5 min. The supernatant was discarded via micropipette, and pelleted spores were subjected to DNA extraction (see below Section 4.2.1).

#### 4.1.3. Preparation of Sampling Rods

Sterilized 304-stainless steel sampling rods (1.1 by 40 mm; Carolina Precision Grinding, Denmark, SC, USA) were coated with silicone vacuum grease (Dow Corning, Midland, MI, USA) and placed in pairs in 2-mL screw cap-tubes. To develop a standard curve for spore quantification and detection limit, pairs of sampling rods were contaminated with known quantities of spores, as described by Villari et al. (2017) [31], and subjected to DNA extraction (see Section 4.2.2).

#### 4.1.4. Preparation of *N. personata* Spores and Fungal Isolates for Use in the Amplification Specificity Test of the qPCR Assay

Late-leaf-spot-symptomatic peanut leaves, approximately 50 each, were collected from nine counties (Allendale, Bamberg, Barnwell, Calhoun, Dorchester, Hampton, Horry, Marlboro, and Orangeburg) in SC, USA in 2018 and 2019. Spores of *N. personata* from each county were prepared for DNA extraction, as described in Section 4.1.2.

Approximately 21 peanut leaves or leaflets were placed on potato dextrose agar (PDA) in separate Petri dishes and incubated at 25 °C under constant fluorescence light for up to 7 days. While arbitrarily taken (without species identification) from each of the placed leaflets, 33 fungal isolates grown out of the 21 peanut leaflets on PDA were selected based on morphological appearance, e.g., colony mycelium color. These 33 fungal isolates (Figure S2) were subjected to DNA extraction (see Section 4.2.3).

### 4.2. DNA Extraction

#### 4.2.1. DNA Extraction Procedures for Use in the Initial Development of the qPCR Assay

DNA was extracted from LLS symptomatic peanut leaves and *N. personata* spores prepared as described previously (see Section 4.1.2) using E.Z.N.A. Plant DS Kit (Omega Bio-tek Inc., Norcross, GA, USA) according to the manufacturer’s protocol. The negative control consisted of DNA similarly extracted from healthy peanut leaves. Extracted DNA was stored at −20 °C.

#### 4.2.2. DNA Extraction Procedures for Analysis of Lab-prepared and Field Collected Sampling Rods

DNA from sampling rods was extracted using a crude DNA extraction (NaOH-based) protocol [40]. The extraction buffer consisted of 10% 1M NaOH, 1% Triton™ X-100 (MiliporeSigma, St. Louis, MO, USA), 0.2% 1M EDTA, and 88.8% sterile water. Rods were aseptically transferred to sterile 2-mL conical base screw-cap tubes containing 100-µL extraction buffer. Tubes were vortexed at 42 Hz for 1 min, immersed in boiling water for 5 min, vortexed again at 42 Hz for 1 min, and boiled for an additional 5 min. Tubes were cooled at room temperature for 5 min before adding 100 µL of 100-mM Tris-HCL buffer pH 2. Tubes were then spun for 3 s, and rods were aseptically removed from the extraction tubes before centrifuging tubes at 12,000× *g* for 3 min. Tubes were stored at −20 °C until used in qPCR assay, which occurred no longer than 2 months after extraction. The supernatant was used as template for qPCR reactions.

#### 4.2.3. DNA Extraction Procedures for Use in the Amplification Specificity Test of the qPCR Assay

DNA of *N. personata* spores and fungal isolates used to test amplification specificity of the primers were extracted using the crude DNA extraction method as described above for sampling rods (see Section 4.2.2). Extraction of DNA from peanut leaflets exhibiting LLS symptoms and non-LLS symptoms was performed using E.Z.N.A. Plant DS Kit (Omega Bio-tek Inc., Norcross, GA, USA) according to the manufacturer’s protocol. Amplification specificity was further evaluated against DNA extracted from three *N. personata* strains collected from Florida in 2016 (Table S1).

### 4.3. Primer Design

The sequences of 18S-28S ribosomal RNA region of *M. berkeleyi* (GenBank accession no. AB435066.1, Kurose et al. 2009 [35] and *N. personata* (GenBank accession no. MF951374.1, Videira et al. 2017) [36] were aligned with the sequences from the same region of all the other *M. berkeleyi* and *N. personata* accessions, as well as 19 other *Mycosphaerellaceae* species available in GenBank using BioEdit version 7.2.6 [44]. Highly conserved regions specific to *N. personata* were used in the design of primers CP5F (5′-TCGGAGTGGTCAAGTAAATTCC-3′) and CP5R (5′-ATATGCCACGCCGCTTAGAGACGG-3′). The primers specifically amplify the Internal Transcribed Spacer (ITS) 2 region in *N. personata* and were evaluated in silico for potential homo-dimer and hetero-dimer with the OligoAnalyzer Tool (Integrated DNA Technologies, Inc., www.idtdna.com). Primers were synthesized by Integrated DNA Technologies, Inc. (Integrated DNA Technologies, Inc., Coralville, IA, USA).

### 4.4. qPCR Reaction Conditions

The optimized qPCR reactions were carried out on a CFX96^TM^Real Time System (Bio-Rad, Hercules, CA, USA) in a total volume of 20 µL containing 1x SsoAdvanced™ Universal SYBR^®^ Green Supermix (Bio-Rad, Hercules, CA, USA), 0.25 µM of each primer, and 1 µL of DNA template. The PCR program consisted of an initial denaturation at 98°C for 3 min followed by 40 cycles of 98 °C for 15 s, 64.2 °C for 20 s, and 72 °C for 30 s, and a melt curve analysis from 65 to 95 °C with an increment of 0.5 °C every 5 s. During each run, a qPCR reaction with no DNA template and a reaction with DNA from healthy peanut leaves were included as negative controls. Data acquisition and cycle threshold (C_t_) analysis were performed using Bio-Rad CFX Manager 3.1 software (Bio-Rad Laboratories, Hercules, CA, USA). Amplification of a single product was evaluated with melt curve analysis and gel electrophoresis on 1.5% agarose gel.

### 4.5. Amplification Specificity of the qPCR Primers

Specificity of the qPCR assay was tested by performing qPCR with DNA of 34 fungal isolates, including 33 isolates obtained from peanut leaves, as described above, and one isolate of *Sclerotium rolfsii*, a causal agent of southern stem rot on peanut (Table S1, Figure S2). Assay specificity was also validated by conducting the qPCR assay with DNA of *N. personata* spores collected from nine different counties in SC, nine peanut leaflets exhibiting non-LLS lesions, and two peanut leaflets with LLS lesions (Table S1, Figure S2). Fungal DNA samples that did not amplify with the CP5F-CP5R primers were further amplified with primers ITS1- ITS4 [45] to verify the presence of fungal DNA. Negative (non-template control and DNA of healthy peanut leaves) and positive controls (DNA extracted from *N. personata* spores) were included in all experiments. In addition to the amplification specificity test using fungal isolates, spores, and peanut leaflets, the specificity of the assay was evaluated by performing the qPCR assay with 27 air biota samples collected from the fields using spore traps prior to planting date (16 April to 22 April) until 5 weeks after planting (22 April to 31 May) (Table S1). The qPCR assay was performed as described above and melt curves were interpreted according to Downey (2014) [33].

### 4.6. Detection Limit of the qPCR Assay

#### 4.6.1. Pure Spore Suspension

The detection limit of qPCR on pure *N. personata* spore suspensions was examined using both purified and crude DNA. DNA from the same number of spores (10, 25, 50, 100, or 1000) used for inoculating rods (see below Section 4.6.2) was extracted using a DNA extraction kit (E.Z.N.A. Plant DS Kit, Omega Bio-tek Inc., Norcross, GA, USA) and the crude DNA extraction method (see above Section 4.2.2). Three independent DNA extractions of each spore concentration were performed using each method. The qPCR assay was performed in triplicate (three technical replications) per DNA sample.

#### 4.6.2. Rod-Borne Spores

Rods were prepared as described above (see Section 4.1.3). To prepare spore suspension, approximately 15 LLS lesions with visually abundant conidiophores were cut and placed in a 2-mL screw cap-tube. Sterile distilled deionized water (500 µL) was added to the tube prior to being vortexed at 42 Hz for 1 min to release spores. The concentration of the spore suspension was estimated using a hemocytometer. Different quantities of spores were pipetted onto separate sampling rod pairs inside sterile 2-mL screw-cap tubes to a final concentration of approximately 10, 25, 50, 100, or 1000 spores per pair of rods [31]. Three independent DNA extractions of each spore concentration were performed using the crude DNA extraction method described above. The qPCR assay was deployed with DNA extracted from artificially inoculated rods with three technical replications per extraction (Table S2). The standard curve was generated by linearly regressing the C_t_ value of each spore concentration from the three independent DNA extractions against log_10_ number of spores and used to estimate the number of *N. personata* spores collected on sampling rods.

### 4.7. Detection of Airborne Inoculum in Peanut Fields

#### 4.7.1. Custom Impaction Spore Trap

**Figure 4:**
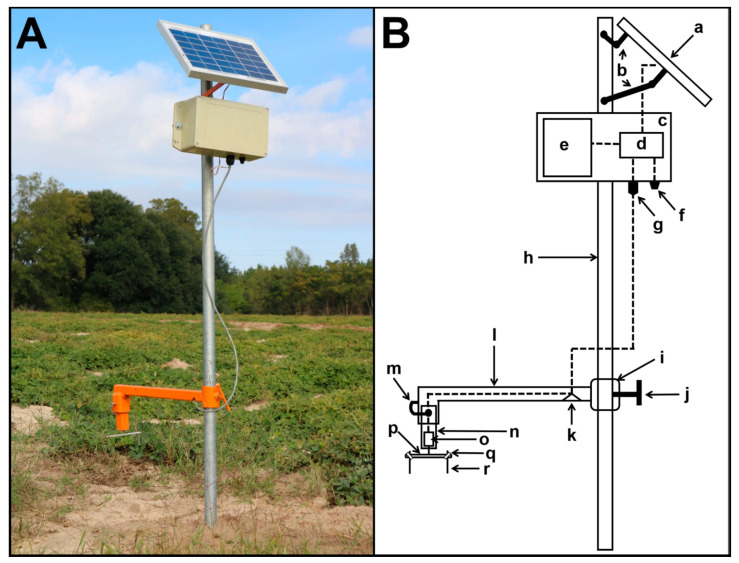
Custom impaction spore trap developed in the study. (**A**) Spore trap placed in the peanut field to sample aerial spores. (**B**) Spore trap components: (**a**) 5-W 6-V solar panel (Solarland^®^ SLP005-06U); (**b**) solar panel holder; (**c**) battery and circuit box; (**d**) voltage regulator circuit board; (**e**) 6-V, 4.5-Ah nonspillable rechargeable SLA battery (Tysonic TY-6-4.5); (**f**) on/off switch; (**g**) panel mount weathertight connector; (**h**) metal pole; (**i**) sampling arm height adjuster; (**j**) height adjustment screw lock; (**k**) cable hole; (**l**) metal arm; (**m**) motor holder lock; (**n**) motor holder; (**o**) motor; (**p**) 4.7 by 90 mm aluminum sampling arm with 40-mm sampling radius; (**q**) rubber o-ring (4.5-mm internal diameter); and (**r**) 1.1 by 40-mm stainless steel sampling rods (Carolina Precision Grinding, Denmark, SC). Dashed lines are Carrol brand 20-gauge two-strand PVC jacketed multiconductor electrical cable (General Cable, Highland Height, KY, USA).

Custom impaction spore traps (Figure 4), modified from Thiessen et al. (2016) [18] and Villari et al. (2017) [31] and similar to the Rotorod Sampler (Sampling Technologies Inc., Minnetonka, MN, USA), were placed one in each of two non-irrigated peanut fields (D13A and D11B) at Edisto Research and Education Center during the 2019 growing season (Figure S3). Spore traps in each field were ~360 m apart (Figure S3). For more than 15 years, D13A was continually planted to peanut but occasionally planted to soybean and had a long history of severe LLS infections. Before 2019, D11B was last planted to peanut in 2016, having been planted to cotton for 2017 and 2018. Adjacent to D11B was a cotton field (Figure S3) where volunteer peanuts from the previous peanut crop year (2018) were abundant at the beginning of the 2019 growing season. In each field, spore trap sampling arms were positioned approximately 15 cm above the ground. The circuit diagram of the voltage regulator used to control motor speed on each spore trap was as previously described by Thiessen et al. (2016) [18]. Each trap was capable of sampling approximately 48.3 ± 1.2 L min^-1^ by spinning two sampling rods at approximately 1 m s^-1^ [18]. Sampling rods were replaced in traps every 2 to 3 days and tested with the qPCR assay. Traps were operated from 16 April to 11 October and from 8 May to 11 October 2019 in D13A and D11B, respectively. Peanut seed was planted in both fields on 23 April 2019.

#### 4.7.2. Weather Data and Disease Incidence

Daily weather data were recorded through the Clemson University, EREC weather station, which is located ~2 km from D13A and D11B. LLS development in two 40-m by 100-m peanut plots (a1 and a2) in D13A and one 30-m by 80-m peanut plot (b2) in D11B (Figure S3) was visually monitored. Incidence of LLS as % symptomatic leaflets in D13A was evaluated on 1, 16, and 29 July, 12 and 26 August, 10 and 23 September, and 10 October. Incidence in D13A was evaluated in both a1 and a2 plots, with the exception of 1 July, 10 and 23 September, and 10 October, where evaluation was conducted only in a1. LLS monitoring in D11B was conducted on 7 and 21 August, 3 and 24 September, and 7 October.

#### 4.7.3. DNA Sequencing of Air Samples with Positive Detection

The qPCR products from two spore trap samples were sent for Sanger sequencing at the DNA Laboratory, School of Life Sciences, Arizona State University. The consensus sequences were retrieved via alignment of the forward and reverse sequences and compared with other sequences in GenBank via BLASTn.

## Supplementary Materials

The following are available online at https://www.mdpi.com/2223-7747/9/10/1327/s1, Figure S1: Melt curve from the qPCR assay with *Nothopassalora personata* DNA. Figure S2: Fungal cultures and symptomatic *Arachis hypogaea* leaflets used for determining specificity of the quantitative Polymerase Chain Reaction (qPCR) assay using primers CP5F-CP5R. **(1–33)** Fungal culture 1–33, **(34)** *Sclerotium rolfsii*, **(35–43)** symptomatic *A. hypogaea* leaflet 1 – 9, and **(44–45)** late leaf spot (LLS) symptomatic *A. hypogaea* leaflet; Figure S3: Schematic representation of the two experimental fields at the Edisto Research and Education Center, Blackville, South Carolina (SC), USA; Figure S4: Magnified bar graph for inoculum detection with estimated spores less than 700 in D13A and D11B; Table S1: Samples used for determining amplification specificity of the qPCR assay using primers CP5F-CP5R; Table S2: Threshold cycle (C_t_) values of qPCR with crude DNA extracted from rods inoculated with different number of spores and controls.
