# Supplementary figures and images for: Early Detection of Airborne Inoculum of Nothopassalora personata in Spore Trap Samples from Peanut Fields Using Quantitative PCR
Source: Plants (Basel). 2020 Oct 9;9(10):1327. doi: 10.3390/plants9101327 (PMC7650562; doi:10.3390/plants9101327)

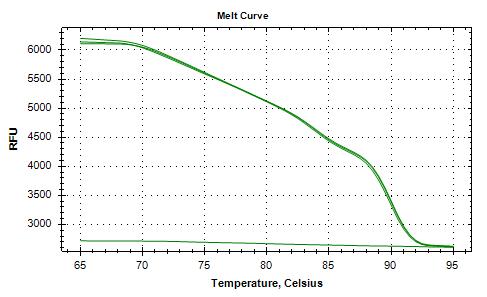

Supplement: Supplementary file 1 [file plants-09-01327-s001.zip › Supplementary Figure S1_no revision.jpg]

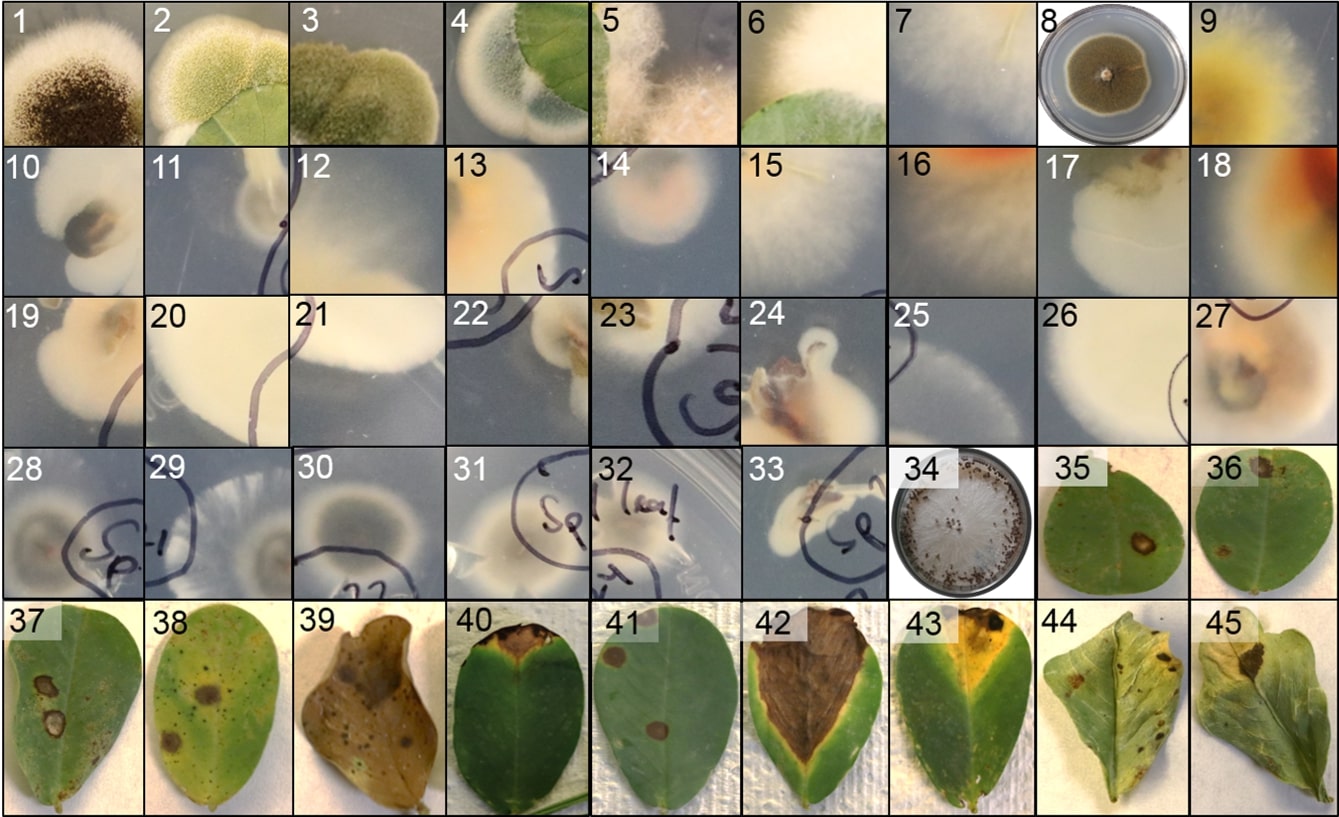

Supplement: Supplementary file 1 [file plants-09-01327-s001.zip › Supplementary Figure S2_no revision.jpg]

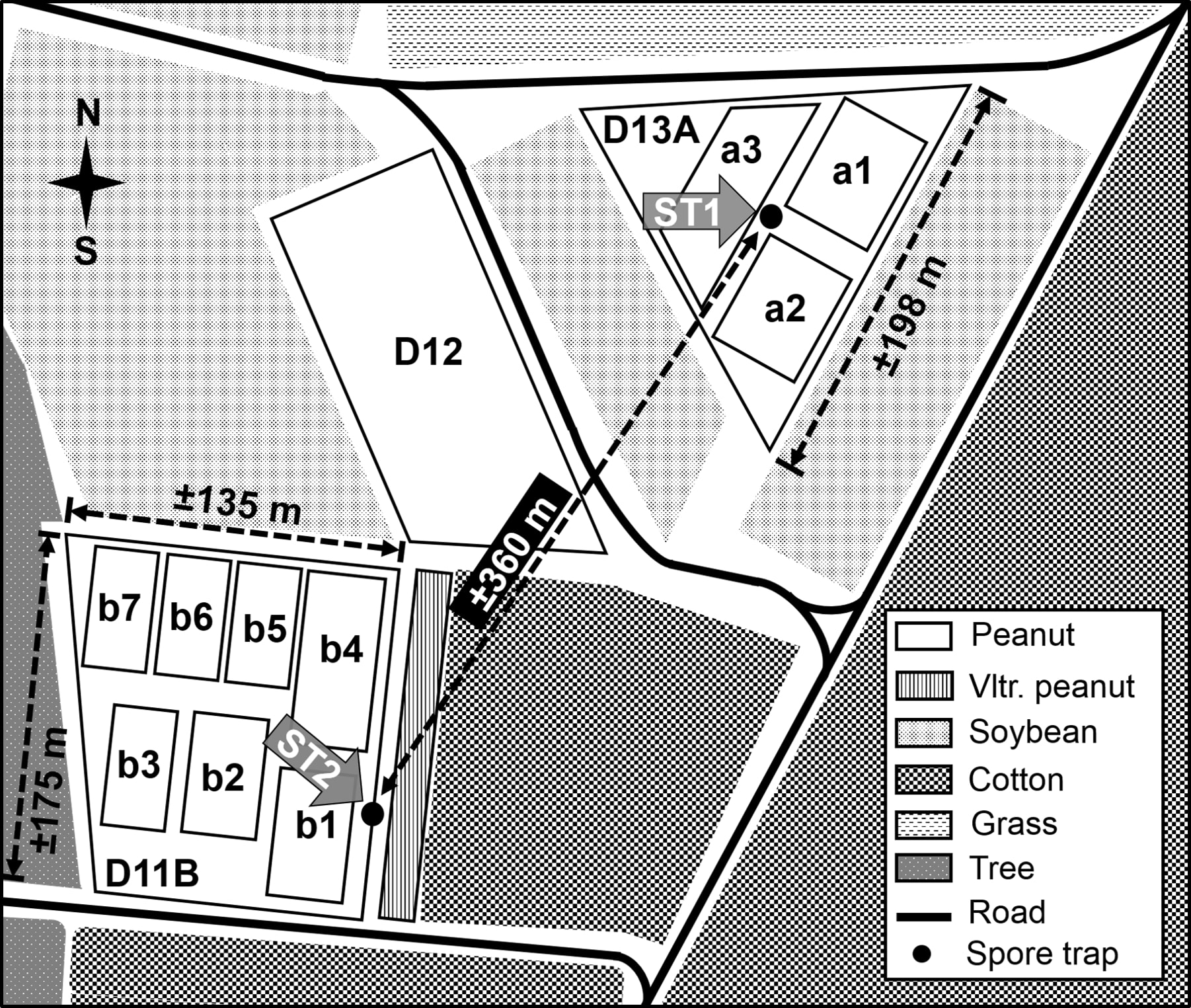

Supplement: Supplementary file 1 [file plants-09-01327-s001.zip › Supplementary Figure S3_no revision.jpg]

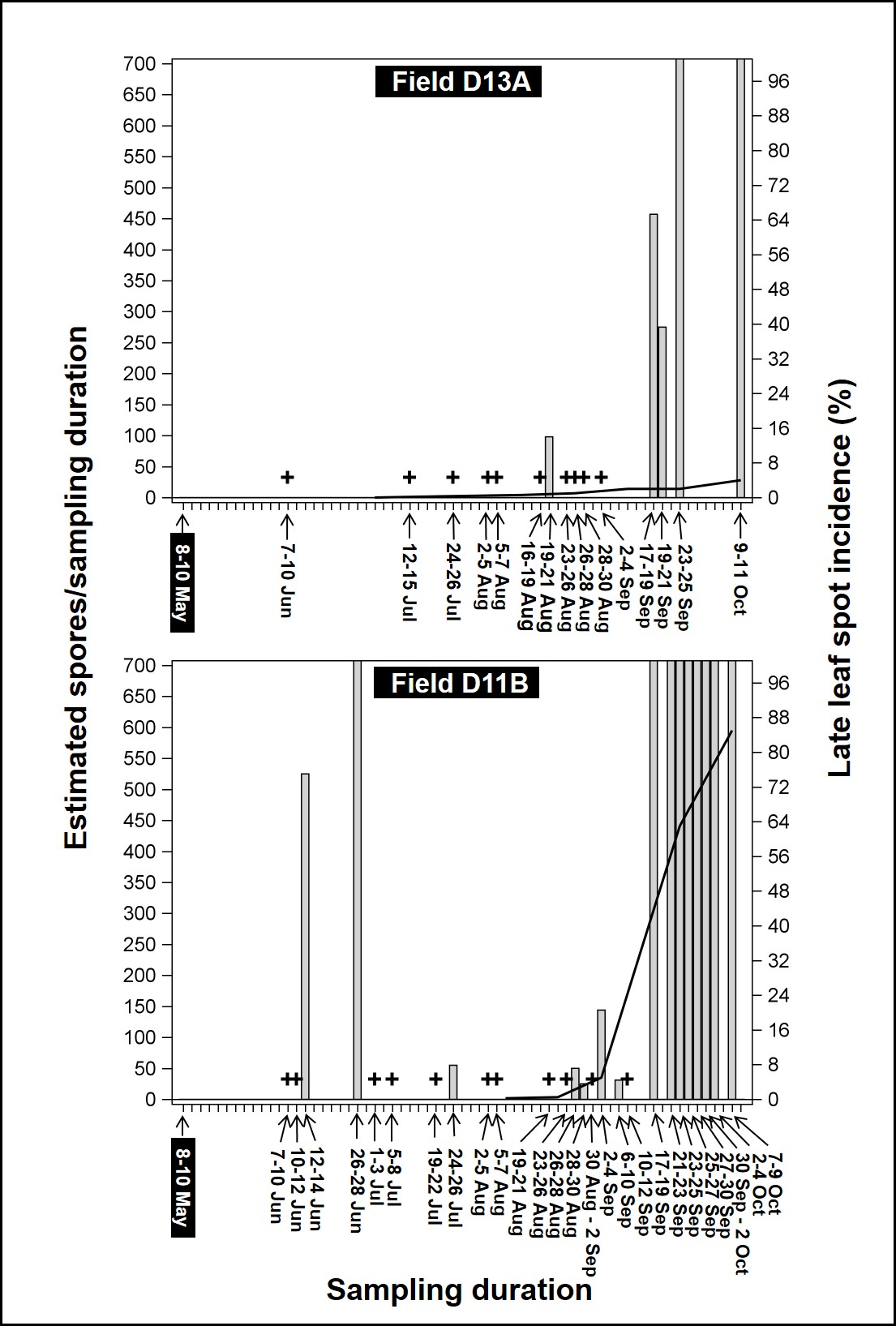

Supplement: Supplementary file 1 [file plants-09-01327-s001.zip › Supplementary Figure S4_the new one_magnified bar graph for spore detection below 700.jpg]
